# Assessment of Urostomy Parastomal Herniation Forces Using Incisional Prevention Strategies with an Abdominal Fascia Model

**DOI:** 10.1016/j.euros.2023.05.019

**Published:** 2023-06-21

**Authors:** Diboro L. Kanabolo, Adam D. Maxwell, Yashwanth Nanda Kumar, George R. Schade

**Affiliations:** a Department of Urology, University of Washington Medical Center, Seattle, WA, USA; b Center for Industrial and Medical Ultrasound, Applied Physics Laboratory, University of Washington, Seattle, WA, USA

**Keywords:** Parastomal hernia, Reconstruction, Ileal conduit, Incision, Bladder cancer, Urostomy

## Abstract

**Background:**

Approximately 10 000 patients undergo cystectomy/ileal conduit annually in the USA, of whom over 70% subsequently develop a parastomal hernia (PSH). Still, no well-established “best” practice for stoma creation to prevent a PSH exists.

**Objective:**

To measure the relationship between incision size/type/material and axial tension force (ATF) as a surrogate for herniation force, using several models to mimic abdominal fascia.

**Design, setting, and participants:**

Abdominal fascia models included silicone membrane, ex vivo porcine, and embalmed human cadaveric fascia. A dynamometer pulled a Foley catheter (20 mm/min) with the balloon inflated to 125% incision (linear, cruciate, and circular) diameter using a motorized positioning system. The maximum ATF before herniation was recorded. The study was repeated in unused silicone/tissue for suture reinforcement. We evaluated silicone, ex vivo porcine, and human abdominal fascia.

**Intervention:**

Incision sizes (1–3 cm) in 0.5-cm increments were evaluated in silicone. A 3-cm incision was used in porcine/human tissue.

**Outcome measurements and statistical analysis:**

ATF for herniation was recorded/compared across incision types/sizes using Mann-Whitney *U* and Kruskal-Wallis tests as appropriate, with *α* = 0.05.

**Results and limitations:**

Linear incision ATF was significantly greater than cruciate and circular incisions. A cruciate incision had significantly greater ATF than a circular incision. In cadaveric tissue, incisions were significantly greater for linear (34.5 ± 12.8 N) versus cruciate (15.3 ± 2.9 N, *p* = 0.004) and for cruciate versus circular (*p* = 0.023) incisions. Results were similar in ex vivo porcine fascia and silicone. Reinforcement with a suture significantly increased ATF in all materials/incision sizes/types. The ex vivo nature is this study’s main limitation.

**Conclusions:**

This study suggests that urostomy fascial incision type may influence ATF required for herniation. Linear incisions may be preferable. Urostomy reinforcement may significantly increase ATF required for a PSH. These data may help establish best practices for PSH risk reduction.

**Patient summary:**

The results of this study illustrate that urostomy fascia incision type may influence the force required to create a parastomal hernia. Linear incisions may be preferable.

## Take Home Message

Ex vivo data suggest that fascial linear incisions are preferable to the more commonly used cruciate incisions. This agrees with the simulation data. Longitudinal versus transverse linear incisions do not make a difference in cadaveric tissue. Suture reinforcement may be effective.

## 1 Introduction

Gold standard definitive management for bacillus Calmette-Guérin refractory or localized muscle-invasive bladder cancer continues to be radical cystectomy (RC) [1]. As part of RC, an ileal conduit is most commonly constructed with a segment of ileum [1]. In the USA, approximately 10 000 patients undergo urostomy construction annually. Of these patients, approximately 85% receive ileal conduit incontinent diversion [1], [2].

Patients with ileal conduits are susceptible to a parastomal hernia (PSH) development, with a prevalence estimated at 60%; PSHs are symptomatic, requiring repair in up to 30% of cases [3]. Despite the high incidence, decades of surgical experience maturing ileal conduits, and morbidity associated with a PSH, no consensus exists on best practices for urostomy creation. Some associations between surgical technical factors and a subsequent PSH have been studied. One such factor is stoma fascial defect size: Hotouras et al. [4] retrospectively described the median abdominal defect diameter for patients with a PSH at 35 mm (range 25–58 mm). For the group without herniation, that number was significantly smaller (22 mm, range 10–36 mm). Another factor is the stoma incision type, which is thought to play a role in pressure differential across a fascial defect, based on the simulation data. Specifically, in multiphysics finite element modeling simulations, cruciate incisions generate higher forces needed for herniation across an incision than circular incisions [5]. Finally, approaches to reinforce the urostomy site may alter the risk of PSH development. Toward that goal, prophylactic mesh is used by some surgeons as the current gold standard for PSH prevention, although there is mixed evidence for its efficacy [6], [7].

Minimal data exist evaluating these aforementioned factors in tissue studies to establish the relationship between incision size/type and the dynamic forces associated with a PSH. We therefore studied the relationship between stoma incision size/type and abdominal wall forces in several ex vivo abdominal wall models and one strategy to reinforce the abdominal wall. An improved understanding of the relationship between urostomy creation techniques and tension forces that cause a PSH and identification of possible prevention strategies in the laboratory will enable the development of improved surgical techniques to reduce the development of a PSH.

## 2 Patients and methods

### 2.1 Models for abdominal fascia

Three models of abdominal fascia were used for the study: silicone membranes, ex vivo porcine fascia, and embalmed cadaveric human fascia.

The 0.5-mm-thickness silicone membranes with a durometer of 50A on the Shore elastomeric or “hardness” scale were purchased (McMaster-Carr, Santa Fe Springs, CA, USA). This model was chosen as it is highly repeatable and has well-characterized properties with ubiquitous usage for nonbiologic human tissue simulation [8]. The membrane was cut into 6.5 × 6.5 cm^2^ sections for all experiments.

Ex vivo porcine fascia was acquired immediately postmortem from unrelated porcine animal studies performed in our laboratory. This model was chosen because previous literature by White and colleagues [9] showed similarities in strength to human fascia. Abdominal wall from porcine subjects was obtained and frozen within 6 h of sacrifice. Subcutaneous fat and muscle were dissected away from fascia and split into 6.5 × 6.5 cm^2^ fascia specimens for use in our experiments. Fascia was thawed on the day of experimentation in 55 °C water bath for 20 min (Fisher Scientific, Hampton, NH, USA).

Embalmed human fascia was obtained through the University of Washington Willed Body Program. Though embalmed muscular and fascial tissues may have different mechanical properties than fresh tissues [10], observation of similar relationships in embalmed human tissues would offer validation of our work/measurements in silicone and porcine tissue. Additionally, it would allow exploration of embalmed tissue as a cost-effective alternative to fresh human fascia in future experimentation. Embalmed tissues were fixed with a solution containing 50% water, 2% formaldehyde, 20% ethanol, 2% methanol, 2% isopropyl alcohol, 4% phenol, and 20% propylene glycol [11]. All tissues were collected from adult cadavers whose demographics were blinded to the research team. Subcutaneous fat, muscle, and skin were again dissected away from the fascia and split into 6.5 × 6.5 × 0.05 cm^3^ fascia specimens.

### 2.2 Fundamental apparatus design

**Fig. 1:**
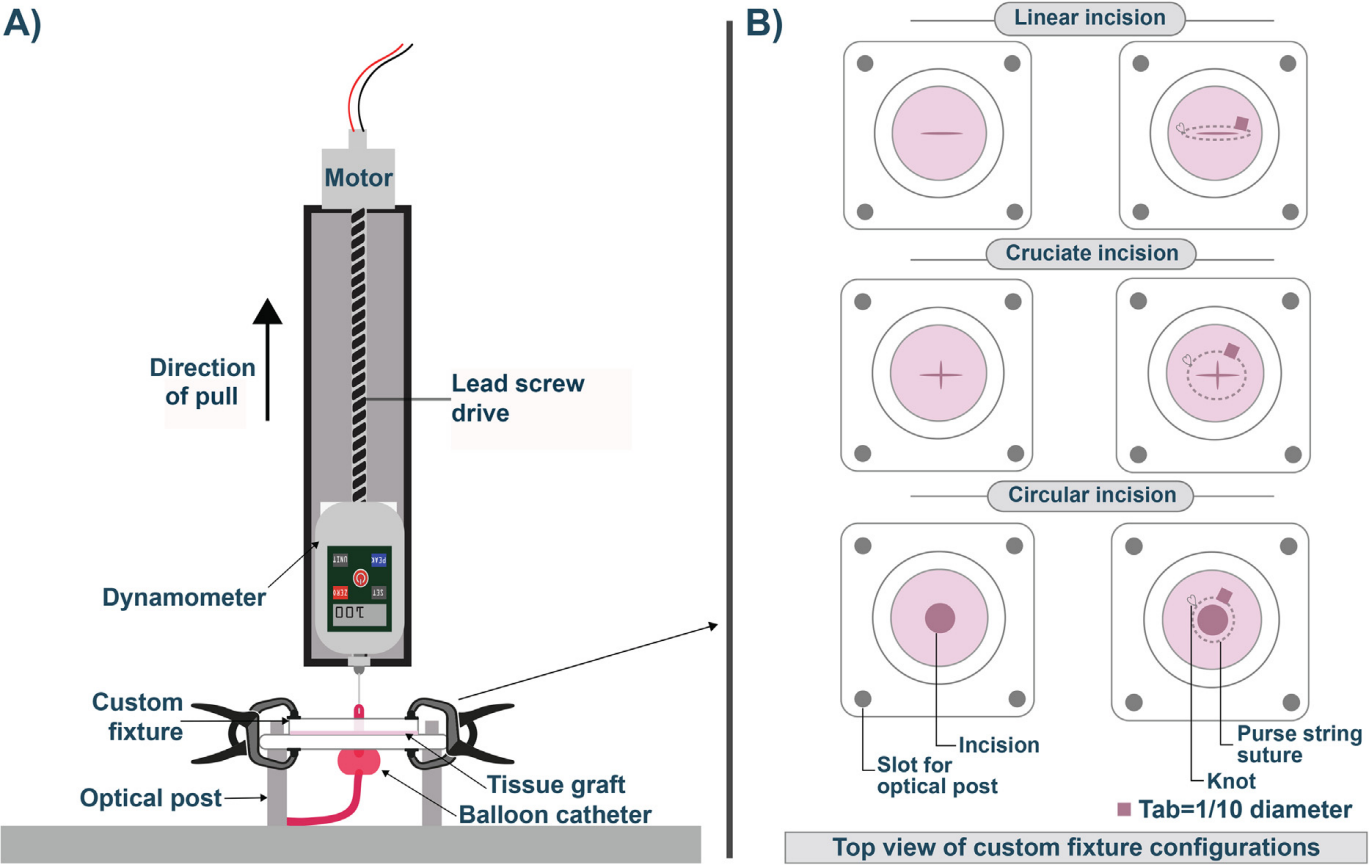
Depiction of experimental apparatus. (A) Illustration of force meter with a hook adapter mounted to a positioning motor to measure the tension force created by traction of Foley balloons across the incision. Balloons were filled with water to 125% of their incision dimension and pulled at 20 mm/min. The peak force in newton before the release of the Foley through the aperture could then be detected. Optical rods were used to mount the custom fixture. (B) Schematic showing a top view of custom fixtures with the three incision types. A scalpel was used to create linear, cruciate, and circular incisions in sheets of silicone and tissue (column 1). We repeated this separately with purse string suture reinforcement (column 2). We used a tab laid at the incision edge to try to prevent constriction of the incision.

To simulate the dynamic forces on the fascial wall, we used axisymmetric indentation of edge-supported soft elastic fascial membranes with a semirigid compressible spherical balloon to herniate across a sheet of silicone or fascia. This was mounted on a custom three-dimensional (3D)-printed fixture (Fig. 1A). A scalpel was used to create linear, cruciate, and circular incisions in silicone “tissue” sheets ranging from 1 to 3 cm in 0.5-cm increments. In porcine/embalmed human fascia, a single sized incision was made for each type due to less availability of tissue. A 3-cm incision was chosen in biologic tissues to simulate the incision size used for the upper limit of normal sized small bowel [12]. Axial tension force (ATF) was measured using a spring-loaded dynamometer capable of traction forces up to 500 N with a precision of 0.1 N (VTSYIQI Instruments, Hefei, China). The dynamometer was mounted to a positioning motor with a hook adapter to measure upward tension force. Following mounting, we attached a 20-French red latex Foley catheter (Bard Medical, Murray Hill, NJ, USA) to the hook at the Foley’s proximal tip. The Foley balloon was hydrated to 125% of the incision dimension being tested. The positioning system was then started and moved in a uniaxial direction at 20 mm/min. The direction of force was perpendicular to the silicone/tissue. The peak force before “herniation” of the balloon through the aperture was detected. Each measurement was repeated for a total of five identical incisions in all tissue types.

To evaluate whether the ATF trend across incision types was consistent when varying the coefficient of friction of the herniating object, we utilized a 3D-printed Foley balloon fitted for 3-cm incision sizes (125% incision diameter) with a simulated drainage aperture. This was composed of 2.85-mm polylactic acid (PLA) filaments constructed with an Ultimaker 3D printer (Ultimaker, Utrecht, The Netherlands). The coefficient of friction for PLA is 0.15–0.28, whereas that for Foley-based rubber is 0.45–0.62 [13], [14].

### 2.3 Suture reinforcement

To evaluate the impact of suture reinforcement of the fascia on ATF of herniation, we repeated the above experiments in virgin silicone/fascia after reinforcing with a monofilament polyamide (nylon) suture. Nylon is a nonabsorbable suture notable for its high tensile strength. Prior data have shown that a suture may bear an increased tensile load, dependent on the material used [9]. A nonabsorbable suture was used due to the ex vivo nature of our experiments. A protracted period was not feasible to confirm any benefit of an absorbable versus a nonabsorbable suture. Following incision, a suture was hand sewn in purse string fashion for ten “throws” approximately 1.5 mm from the incision edge. A plastic tab of 10% of incision diameter was laid while tying a suture to prevent incisional constriction (Fig. 1B). A size 4-0 nylon monofilament suture was first used in silicone as a proof of principle, followed by porcine fascia. These experiments were repeated in embalmed cadaveric tissue using a slightly larger 3-0 nylon suture for reinforcement. A larger suture was used because embalmed tissue introduces collagen cross-links that slightly increases the stiffness of the cadaveric tissue and require higher torque on the suture needle for placement, that is, to prevent tearing of the tissue from a smaller needle/suture during the placement of the suture [10].

### 2.4 Linear incision subgroup analysis

**Fig. 2:**
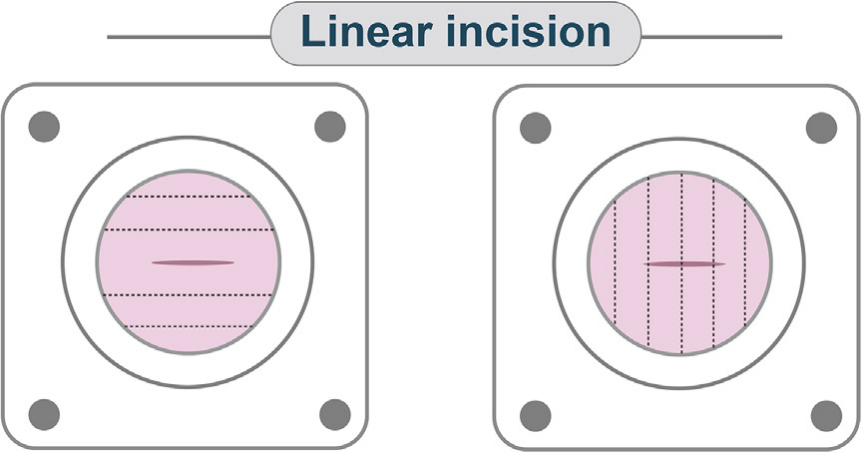
Longitudinal versus transverse incision. Within cadaveric tissue, to evaluate the effect of linear incision direction, both longitudinal and transverse incisions were created and compared. The black orientation lines are used to indicate horizontal versus vertical nature of the tissue with respect to the incision.

To determine the impact of natural abdominal fascial lines on ATF with respect to linear incisions, a longitudinal vertical incision was made and compared with a transverse incision for an adjacent fascial specimen (Fig. 2).

### 2.5 Statistical analysis

We treated silicone specimens as independent with equal variance due to material homogeneity. Porcine or cadaver specimens were independent with unequal variance given tissue heterogeneity. The Mann-Whitney *U* test was used to compare between two groups, and the Kruskal-Wallis test was used to compare between three groups due to nonparametrical distribution (GraphPad Prism, San Diego, CA, USA). An a priori sample size of *n* = 5 for each variable was used as it was determined to yield an 80% statistical power to detect a 0.9 MPa (90 N/cm^2^) difference in pressure between groups (eg, comparisons between incision types for any constant cross-sectional incision size) with alpha = 0.05, assuming a standard deviation of 20% of the mean.

## 3 Results

**Fig. 3:**
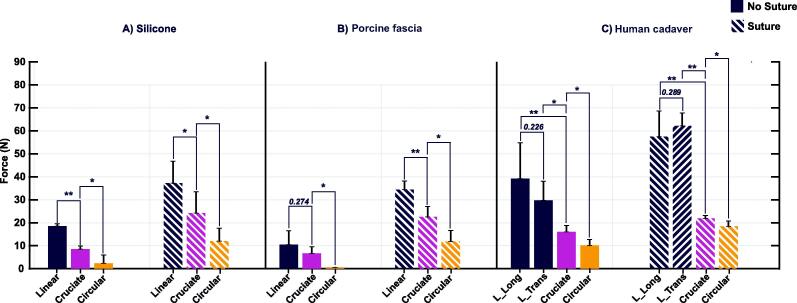
Axial tension force (in newton) of herniation across incision types. Data are depicted stratified by tissue type: (A) silicone membrane, (B) fresh porcine fascia, and (C) embalmed human fascia. Suture reinforcement provided significantly greater force needed for herniation than nonsutured silicone, porcine fascia, and human cadaver. L_long = linear longitudinal; L_trans = linear transverse. *p < 0.05. **p < 0.01.

Incision size, type, and presence of suture had a significant effect on the ATF for herniation in silicone. The mean ATF for incisions ranging from 1 to 3 cm is noted in Table 1. Linear incisions were associated with significantly higher ATF than cruciate or circular incisions at all incision sizes. Likewise, cruciate ATF was significantly higher than circular incision ATF for all sizes (Fig. 3A). Suture reinforcement led to significantly higher ATF of herniation for all incision types tested, and the relationships between incision types remained similar.

**Table 1:** Axial tension forces of herniation in silicone membrane stratified by incision size and type

Silicone axial tension force					
	Size (cm)	Force (N), mean (SD)			*p* value
No suture		Linear	Cruciate	Circular	
	1	5.3 (1.2)	3.3 (2.4)	0.0 (0.0)	0.0018
	1.5	8.8 (0.9)	5.0 (2.1)	0.6 (1.3)	<0.0001
	2	13.1 (3.5)	7.9 (2.6)	0.8 (1.8)	0.0002
	2.5	16.0 (2.0)	8.3 (2.1)	1.4 (1.9)	<0.0001
	3	18.6 (1.0)	8.5 (1.4)	2.4 (3.6)	0.0001
Suture					
	1	10.4 (1.9)	8.2 (2.7)	3.5 (0.6)	0.0013
	1.5	18.2 (3.1)	9.4 (3.4)	7.6 (1.4)	0.0012
	2	22.9 (1.1)	10.2 (1.2)	7.2 (1.7)	<0.0001
	2.5	26.8 (3.1)	15.6 (6.8)	8.9 (3.5)	0.0033
	3	37.2 (9.5)	24.2 (9.3)	12.0 (5.6)	0.0014

SD = standard deviation.

Kruskal-Wallis test was used for the evaluation of statistical significance.

To evaluate friction’s effect on these results, ATF was also measured in silicone membranes with low-friction 3D-printed PLA balloons. Table 2 provides the ATF needed for the herniation of a 3D-printed PLA balloon through silicone membrane for all incision types of 3-cm diameter. The mean force ± standard deviation (SD) significantly declined from linear (19.2 ± 0.5 N) to cruciate (7.6 ± 0.4 N) and from cruciate to circular (3.7 ± 0.1 N) incisions, with a constant 3-cm incision size (*p* < 0.0001).

**Table 2:** Comparative ATF needed for herniation through silicone membrane using a three-dimensional printed Foley catheter balloon with PLA filament

Unreinforced axial tension force at 3.0 cm: low friction		
Incision type	Force (N), mean (SD)	*p* value
Linear	19.2 (0.5)	<0.0001
Cruciate	7.6 (0.4)	
Circular	3.7 (0.1)	

ATF = axial tension force; PLA = polylactic acid; SD = standard deviation.

Kruskal-Wallis test was used for the evaluation of statistical significance.

Ex vivo porcine fascia (Fig. 3B) overall performed similarly to silicone specimens with 3-cm incisions. The mean ± SD ATF in porcine fascia was significantly different (*p* = 0.001) across nonsutured linear, cruciate, and circular incisions (10.4 ± 6.0, 6.7 ± 2.9, and 0 ± 0 N, respectively). Both linear and cruciate incision ATF were significantly greater than circular incisions. Following suture reinforcement, the mean ± SD ATF was significantly greater (*p* < 0.0001) than in case of no reinforcement for all incision types (34.4 ± 3.8, 22.6 ± 4.5, and 11.8 ± 4.8 N for linear, cruciate, and circular incisions, respectively).

Ex vivo human cadaveric fascia (Fig. 3C) also performed similar to silicone specimens with 3-cm incisions. The mean ± SD ATF in cadaveric fascia was significantly different (*p* = 0.004) across linear, cruciate, and circular incisions (34.5 ± 12.8, 16.0 ± 2.8, and 10.1 ± 2.5 N, respectively). Both linear and cruciate incision ATF were significantly greater than circular incisions. Following suture reinforcement, the mean ± SD ATF was significantly different (*p* = 0.0018) for all incision types (59.8 ± 8.7, 21.9 ± 1.3, and 18.4 ± 2.4 N for linear, cruciate, and circular incisions, respectively). We further compared linear longitudinal (vertical) versus transverse incisions of 3 cm size to determine whether fascial incision direction affected herniation ATF. No statistically significant differences were noted between longitudinal and vertical incisions in either nonsutured (*p* = 0.226) or sutured (*p* = 0.289) tissue.

## 4 Discussion

We evaluated the relationship between stomal fascial incision factors and ATF in several abdominal wall models to better understand how the surgical technique may impact the risk of a subsequent PSH following ileal conduit. We observed that incision shape, size, and suture reinforcement were all significantly associated with ATF of herniation, an effect that persisted, irrespective of the model material studied. Our data suggest that a linear incision is preferred to a cruciate incision, and a cruciate incision is preferred to a circular one. This agrees with the simulation literature, as Ambe [5] suggested an increase in pressure differential for cruciate compared with circular incisions.

Although general trends between linear, cruciate, and circular incisions were consistent with other tissues, a statistically significant difference was not noted in nonsutured porcine tissue linear versus cruciate incisions. We observed that the ATF demonstrated in nonsutured porcine tissue was of the lowest range (0–10 N) among the three materials studied. The force range was sufficiently low to be overcome by a proportionally higher mean SD between the two incision types. When suture reinforced, the statistical differences were revealed between the two (Fig. 3B).

We also assessed the importance of incision directionality. Abdominal fascial fibers lie transversely, and prior literature has postulated that suture repair of vertical incisions is weaker, relative to transverse incisions, with higher rates of evisceration and incisional hernia [15]. Our study did not reveal a statistically significant difference between longitudinal and transverse incision ATF. The use of internal control with fascia for both incision types obtained within the same cadaver specimen reasonably suggests that fascial variability was unlikely to bias our results. We also note that no difference was seen in ATF between the sutured transverse versus longitudinal incision types.

Extrapolating these findings, our data suggest that linear incisions are preferable over cruciate incisions, and cruciate incisions are preferable to circular removal of abdominal fascia for any given size incision.

It is a unique challenge to simulate bowel herniation, and we are limited by the ex vivo nature of the study. We also acknowledge the variations in total cross-sectional area between incision types. Once deformed, a linear incision creates a narrow ellipse. Unlike the linear incisions, a circular incision has a pre-existing defect. In our study, for a linear incision, a very narrow ellipse after deformation is created just prior to herniation (eg, 3.0 × 0.3 cm^2^ in the *x*-*y* dimensions [area of ∼0.7 cm^2^]). A 3-cm-diameter circle deformed an additional 0.3 cm in 8.5 cm^2^ in cross-sectional area. To create a similar cross-sectional surface area, either the 3-cm linear incision must be lengthened to 9 cm or the circular incision must be decreased to approximately 1.8 cm in diameter. However, this creates clinical constraints, as we have established that we are limited by a small bowel of 3 cm [12]. Therefore, for consistency of analysis and clinical fidelity, we opted to compare incision lengths with the same diameter in the maximum dimension. Additionally, we postulated that friction with some models may play a role in the force needed for herniation, as evidenced by increased ATF for 3- versus 1-cm incisions when controlling for incision shape and reference balloon diameter at 125% of incision size. We suspect that the effect of increased force with increased size is due to friction, for which the silicone membrane is most sensitive due to its relatively higher coefficient of friction [16]. Notably however, the trend for changes in force of herniation across incision types is consistent when varying the sheet material held in the custom fixture or the balloon material. Isolation of the fascia for the purposes of this mechanistic study provides helpful information about the differences in ATF for bowel within our study design. Another potential limitation is the use of embalmed cadaveric fascia, which is modified by tissue fixation, introducing collagen cross-linking and decreasing the elasticity of the specimen. Despite this limitation, the observations in embalmed cadaveric fascia were similar to silicone membrane and porcine fascia, suggesting that embalmed fascia may be an appropriate cost-effective model as we transition to clinically optimized models of human tissues.

It must be acknowledged that the nature of the ex vivo study creates some difficulty in differentiating statistical from clinical significance. Cobb and colleagues [17] performed urodynamic studies in healthy adult volunteers, noting that the intra-abdominal pressure reached a mean of 252 mmHg during stress maneuvers (cough, lift) [18]. This correlated with an abdominal wall tensile force of 27 N/cm [18]. Mesh materials used for herniation have a tensile strength between 16 and 59 N/cm [18], [19]. Our force results are comparable. Preclinical in vivo and clinical studies must be performed to confirm the safety and efficacy of an absorbable versus a nonabsorbable suture.

## 5 Conclusions

This ex vivo study suggests that incision type and suture reinforcement have predictable influences on ATF for herniation, and linear incisions appear to be preferable. These data may bolster the case to standardize urostomy creation, reducing PSH risk.

  ***Author contributions:*** Diboro L. Kanabolo had full access to all the data in the study and takes responsibility for the integrity of the data and the accuracy of the data analysis.

  *Study concept and design*: Kanabolo, Nanda Kumar, Maxwell, Schade.

*Acquisition of data*: Kanabolo.

*Analysis and interpretation of data*: Kanabolo.

*Drafting of the manuscript*: Kanabolo, Nanda Kumar, Maxwell.

*Critical revision of the manuscript for important intellectual content*: Maxwell, Schade.

*Statistical analysis*: Kanabolo.

*Obtaining funding*: Kanabolo, Maxwell, Schade.

*Administrative, technical, or material support*: Kanabolo, Maxwell, Schade.

*Supervision*: Kanabolo, Nanda Kumar, Maxwell, Schade.

*Other*: None.

  ***Financial disclosures:*** Diboro L. Kanabolo certifies that all conflicts of interest, including specific financial interests and relationships and affiliations relevant to the subject matter or materials discussed in the manuscript (eg, employment/affiliation, grants or funding, consultancies, honoraria, stock ownership or options, expert testimony, royalties, or patents filed, received, or pending), are the following: None.

  ***Funding/Support and role of the sponsor:*** This work was supported in part by the 2022 Urology Care Foundation Residency Research Award (American Urological Association) and the Leadership in Education, Achievement and Diversity (LEAD) Program, funded by sponsor Urovant Sciences, Inc.
